# Multimodal force and temperature tactile sensor based on a short-channel organic transistor with high sensitivity

**DOI:** 10.1038/s41598-023-43360-y

**Published:** 2023-09-27

**Authors:** Antonello Mascia, Andrea Spanu, Annalisa Bonfiglio, Piero Cosseddu

**Affiliations:** 1 Department of Electrical and Electronic Engineering, University of Cagliari, via Marengo, Cagliari, 09123 Italy; 2 Department of Science, Technology and Society, Scuola Universitaria Superiore IUSS, Palazzo del Broletto, Piazza della Vittoria 15, Pavia, 27100 Italy

**Keywords:** Engineering, Electronic devices, Sensors and biosensors

## Abstract

In this manuscript, we report on a novel architecture for the fabrication of highly sensitive multimodal tactile transducers, for the simultaneous detection of temperature and force. Such devices are based on a flexible Organic Charge Modulated Field Effect Transistor (OCMFET) coupled with a pyro/piezoelectric element, namely a commercial film of poly-vinylene difluoride (PVDF). The reduction of the channel length, obtained by employing a low-resolution vertical channel architecture, allowed to maximize the ratio between the sensing area and the transistor’s channel area, a technological approach that allows to considerably enhance both temperature and force sensitivity, while at the same time minimize the sensor’s dimensions. Thanks to the employment of a straightforward, up-scalable, and highly reproducible fabrication process, this solution represents an interesting alternative for all those applications requiring high-density, high-sensitivity sensors such as robotics and biomedical applications.

## Introduction

In the last 15 years, the rise of highly flexible materials and electronic systems dramatically changed the biomedical landscape. In fact, novel approaches and solutions in the fields of flexible, soft and epidermal electronics, lead to the introduction of innovative wearable devices for the monitoring of different kinds of biosignals, as well as high-performing passive and active materials for artificial biomimetic muscles, soft neural implants and smart or minimally invasive surgical tools^1–4^. In particular, within the context of sensing and biosensing, as opposed to conventional, rigid, silicon-based electronics, flexible electronics allows the fabrication of systems based on polymeric materials, which can provide outstanding mechanical properties, thus enabling unprecedented applications in challenging fields such as the biomedical and the robotic, where high sensitivity and adequate spatial resolution are usually strict requirements. Possible examples are the so-called electronic-skin systems (e-skin^5,6^), meant as large area surfaces endowed with arrays of devices able to transduce a variety of parameters to mimic the functions of the human skin, wearable patches for the reconstruction of biosignals spatial maps^7,8^, ultra-conformable arrays of force and pressure sensors for electronic skins and haptics^9,10^, wearable mechanical sensors for the monitoring of bio-mechanical parameters such as breath rate, internal pressure, joints motion, and posture^11–14^, as well as temperature sensors for wearable and electronic skin applications^15,16^. Especially in the field of tactile sensing, the need of highly flexible mechanical, force/pressure, and temperature transducers lead to several interesting approaches and solutions^17–21^. However, very few examples of integrated multimodal sensors, capable to detect at the same time different stimuli have been reported so far.

An interesting solution to this important requirement is represented by the work of Zhang et al.^22^, who reported about the development of flexible dual-parameter temperature–pressure sensors based on microstructure-frame-supported organic thermoelectric (MFSOTE) materials. The device is able to transduce both temperature and pressure stimuli into two independent electrical signals, thus permitting the instantaneous sensing of temperature and pressure with a temperature resolution of 0.1 K and a pressure sensitivity up to 28.9 kPa^−1^. More recently, Shin et al. reported about the employment of an interlocked microstructure of polarity-modulated ferro-electric thin films, capable to detect at the same time temperature and pressure^23^. The device exploited both the triboelectric and pyroelectric effects of the ferroelectric microstructures that enable the simultaneous detection of mechanical and thermal stimuli in a single device. The multimodal tactile sensor provides ultrasensitive pressure and temperature detection capability (2.2 V·kPa^−1^, 0.27 nA·°C^−1^) over a broad range of 0.1–98 kPa for the pressure and − 20  to  30 °C for the temperature. A similar structure, still based on chemically modified piezoelectric thin films, have been reported by Fastier-Wooller et al. for the fabrication of tactile sensors for robotic applications capable of discriminating between dynamic and static pressure events^24,25^, while Park and co-authors^26^ developed a fingertip skin–inspired microstructured ferroelectric skins for the detection of static/dynamic pressure stimuli and temperature variations. Recently, You et al. have developed a deformable ionic-based receptor that can distinguish simultaneously spatial profiles of temperature and strain without signal interference^27^. From the analysis of the ion relaxation dynamics, the authors can derived simultaneously strain and temperature vales. Particularly, the normalized capacitance (C/C_0_) is the temperature-insensitive extrinsic variable related to the strain, while the charge relaxation time (τ)) is the strain-insensitive variable to measure the absolute temperature. The authors demonstrated that the strain measurement (C/C_0_) has a linear response in the range between 0 and 50%, while the temperature related signal ln(τ) is used to detect the temperature without geometrical information of the sensor. The temperature sensitivity was 10.4% per °C. Recently, Wang et al.^28^ proposed a self-powered multimodal pressure/temperature tactile sensor based on flexible Bi–Te thermoelectric film and porous microconed elastomer. The authors showed that the integration of the two films allows a multimodal measurement. Particularly, the Bi–Te based thermoelectric film enables the sensor to transduce the temperature with high resolution (< 0.1 K) and with an excellent sensitivity of 3.77 mV·K^–1^. Meanwhile, the porous microconed elastomer responds to the pressure variations, with low-pressure detection (16 Pa) and a high sensitivity of 37 kPa^–1^.

While there exist many publications of two terminal devices for multimodal sensing, very few works have been reported on the employment of flexible transistor architectures to tackle the same goal. In fact, the employment of a transistor structure for the realization of a sensing system has several advantages. First of all, the transduction mechanism can be modulated and locally amplified by the vertical gate field. Moreover, when arrays or matrices of transducers are required, which is actually the case when developing artificial skin systems, the switching capabilities of the transistor can allow to dramatically reduce the overall system design complexity.

Very interesting implementations of this approach have been proposed by Someya’s group, who developed one of the earliest example of a multimodal pressure/temperature sensor base on an organic thin film transistor^29^. Other examples are those developed by Lee and co-workers, who studied different organic multimodal transistor-based sensors including infrared light, strain, temperature and pressure transducers^30,31^, where the key component was the highly crystalline co-polymer P(VDF-TrFE), integrated directly into the OFETs as a multi-functional gate dielectric layer with piezoelectric and pyroelectric characteristics. More recently Meng et al. developed a pentacene-based organic thin film transistor integrated with a piezoelectric ceramic for the realization of a dual mode pressure–temperature sensor^32^. In their work, the authors showed the relationship between the mobility and temperature with a linear response of the device to the temperature change in the range from 20 to 60 °C. Furthermore, once pressure stimulation was applied to the device, the output current varied accordingly. With the intention of obtaining a high-sensitive integrated and compact transducer, in this work, we report on a multimodal tactile sensor with enhanced sensitivity and reduced size based on an organic charge modulated field effect transistor^33,34^, and a vertical structure that allows to easily reduce the dimension of the transistor^35^. The peculiar structure of the device, in fact, allows to tune the sensitivity by modifying the device geometrical parameters^36^, while the vertical structure allows to obtain short-channel, miniaturized organic transistors using an up-scalable and highly reproducible fabrication process. By coupling this short-channel charge sensor and a pyro/piezoelectric sensing element, we have obtained a multimodal tactile sensor able to transduce simultaneously temperature and force variations, with enhanced sensitivity and reduced size, thus offering an interesting alternative and convenient solution for all those applications requiring high device density and high sensor sensitivity.

## Materials and methods

**Figure 1:**
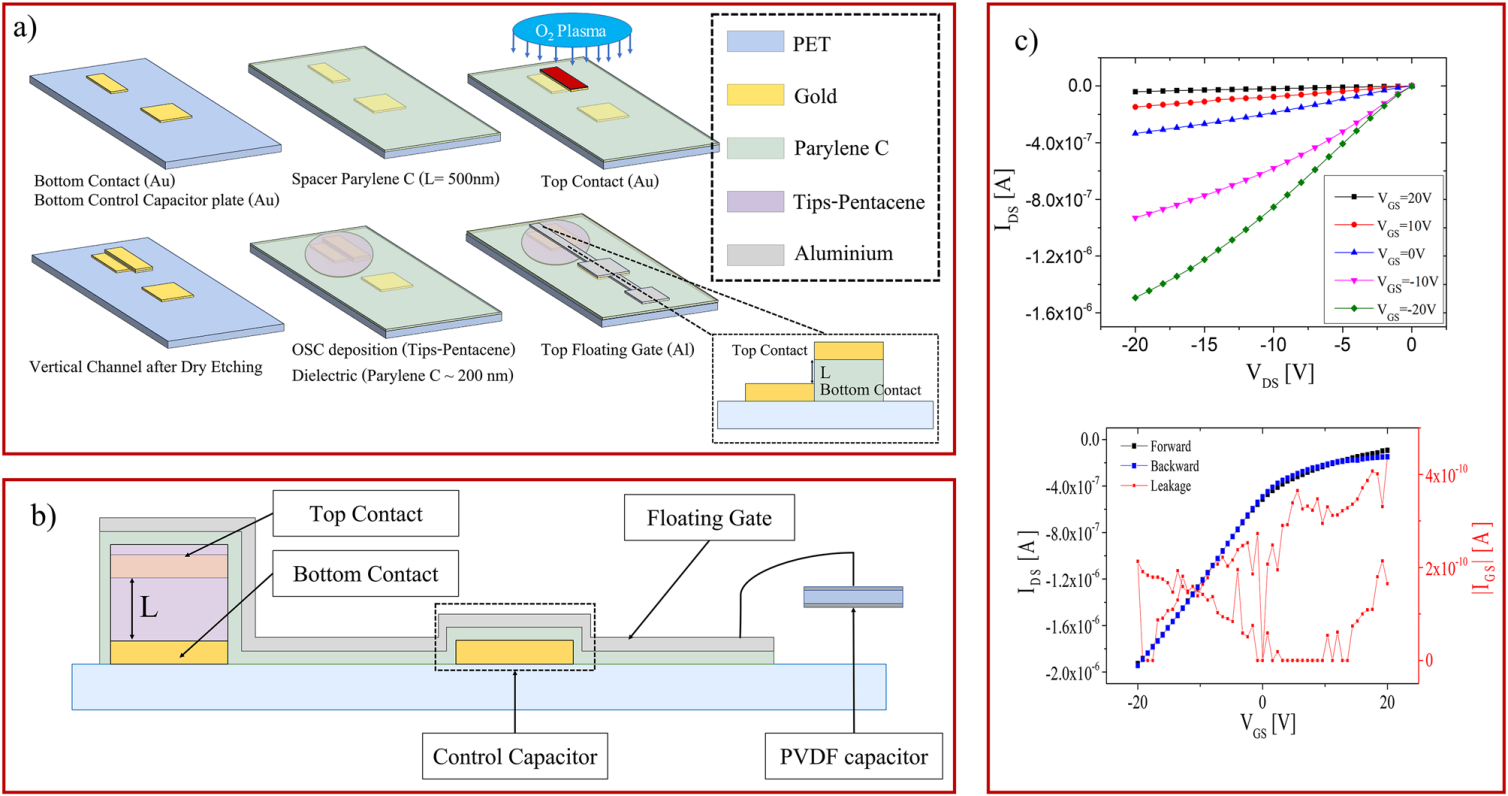
(**a**) Fabrication process workflow and employed material for the sub-micrometer vertical channel OCMFET. In the inset, the transferal cross-section through the vertical channel is reported; (**b**) longitudinal cross-section of the proposed device, where the vertical channel is highlighted. The control capacitor is obtained by overlapping the top floating gate and a gold bottom plate. The sensing area of the sensor is connected to the PVDF capacitor for the temperature and mechanical characterization; (**c**) electrical characterization with output and input characteristics of the sub-micrometer vertical channel OCMFET for tactile sensing. The device shows no hysteresis and low leakage current.

All the devices presented in this work have been fabricated on a 175µm-thick poly(ethylene terephthalate) (PET) substrate. The first step consists in the deposition and patterning, by means of a standard photolithographic process of two gold contacts, i.e. the bottom contact that will act as source of the transistors and the bottom plate of the control capacitor. Afterwards, a Parylene C spacer has been deposited on the whole substrate by chemical vapor deposition (CVD) at room temperature: its thickness defines the channel length of the transistor (500 nm for all the devices in this work). On top of the spacer, a second gold contact (top contact) is deposited and then patterned by a standard photolithography process, thus defining a channel width of 1 mm. Bottom and top contacts will act as source and drain electrodes of the transistor. In order to create the vertical channel, the substrate is exposed to oxygen plasma, which removes the Parylene C from everywhere but under the top contact, which acts here as a mask, thus creating a sub-micrometric step-edge structure that defines the channel of the final organic transistor. Subsequently, a droplet of a TIPS-pentacene solution in anisole (1 wt%) is deposited directly over the transistor channel and let dry at 70 °C on a hot plate. Afterwards, a 200 nm-thick film of Parylene C, acting as gate dielectric, is deposited through CVD, followed by the deposition of the aluminum top floating gate, which has been thermal evaporated and then patterned through a standard photolithographic process. The floating gate presents an elongated design aimed to overlap the bottom plate of the control capacitor (which act as gate terminal in the structure) and it ends with the so-called sensing area. Figure 1a, b show the device fabrication process and the device cross-section, respectively.

Poly(vinylidene fluoride) (PVDF) capacitors have been fabricated using already stretched and poled 28 μm-thick PVDF films from Measurement Specialties Inc.-MEAS. Two thermal evaporated aluminum electrodes have been realized on both sides of the film through a shadow mask and cut in different dimensions (1, 0.5 and 0.25 cm^2^). In the integrated device, the capacitor has been attached on the sensing area of the sub-micrometer vertical channel OCMFET using a couple of droplets of silver ink. After that, the capacitor has been pressed and let dry into an oven at 30 °C for one hour.

## Results

**Figure 2:**
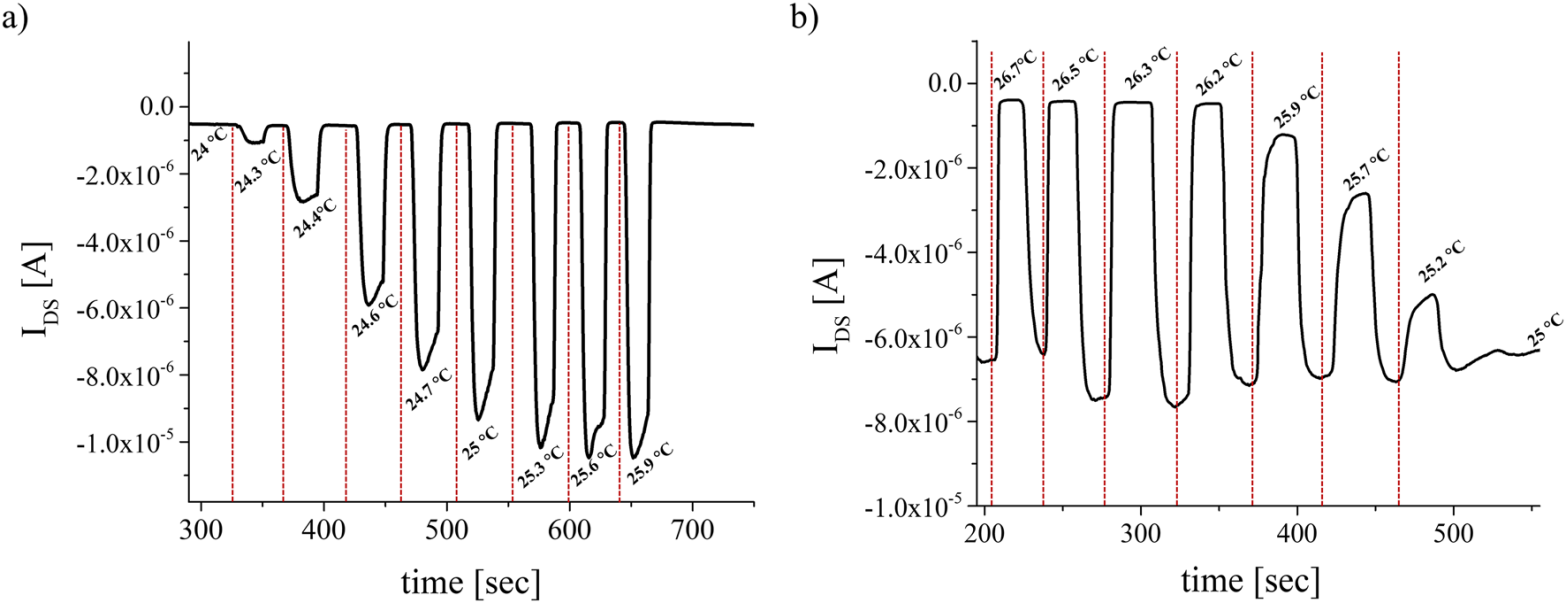
Flipping test of the Vertical-channel OCMFET. (**a**) The sensor output current increases as the temperature increases; (**b**) Opposite sensor response, i.e. a decrease of the output current, while the temperature is increased, due to the flipped connection of the PVDF capacitor onto the floating gate.

At first, all the fabricated vertical sub-micrometer channel OCMFETs have been electrically characterized by connecting the source electrode to the ground of the acquisition system, and applying a voltage drop between the drain electrode and the source (V_DS_). The gate voltage (V_GS_) has been applied between the control capacitor and the source. An example of the typical output and transfer curve are reported in Fig. 1c. It is worth mentioning that due to the self-encapsulating architecture, the device shows very good electrical behavior even after several months, as reported in Figure SI 2 in the Supporting Information. Afterwards, the devices have been characterized as temperature sensor. As said, in this case, the top electrode of the PVDF capacitor is connected through a wire on the sensing area of the OCMFET, while the PVDF capacitor bottom plate is connected to the transistor source electrode. The employment of this solution allows to mechanically decouple the sensing area of the OCMFET to the device channel, thus making more efficient the characterization of the sensor’s response. In order to exercise the temperature stimuli only on the PVDF capacitor, thus exploiting the pyroelectric response, the bottom plate of the capacitor is placed on the top of a Peltier cell, which is powered by a benchtop voltage power supplies, and the overall temperature is controlled by means of an infrared Temperature Sensor (PyroCouple Calex PC21MT-1, Calex electronics limited). As reported in Fig. 2a, an increase of the temperature creates a charge separation, due to its pyroelectricity, into the PVDF film, that perturbates the floating gate charge, thus giving rise to an increase of the output current. Interestingly enough, when the top plate is connected to the floating gate, see Fig. 2b, an increase of the temperature determines a decrease of the output current. This phenomenon is consistent with the fact that in such case opposite charges are induced into the floating gate, thus shifting the device threshold voltage towards more negative values. These flipping tests are necessary to demonstrate that the current variations are actually induced by the pyroelectric effect of the PVDF capacitor and not by some random undesired effect.

**Figure 3:**
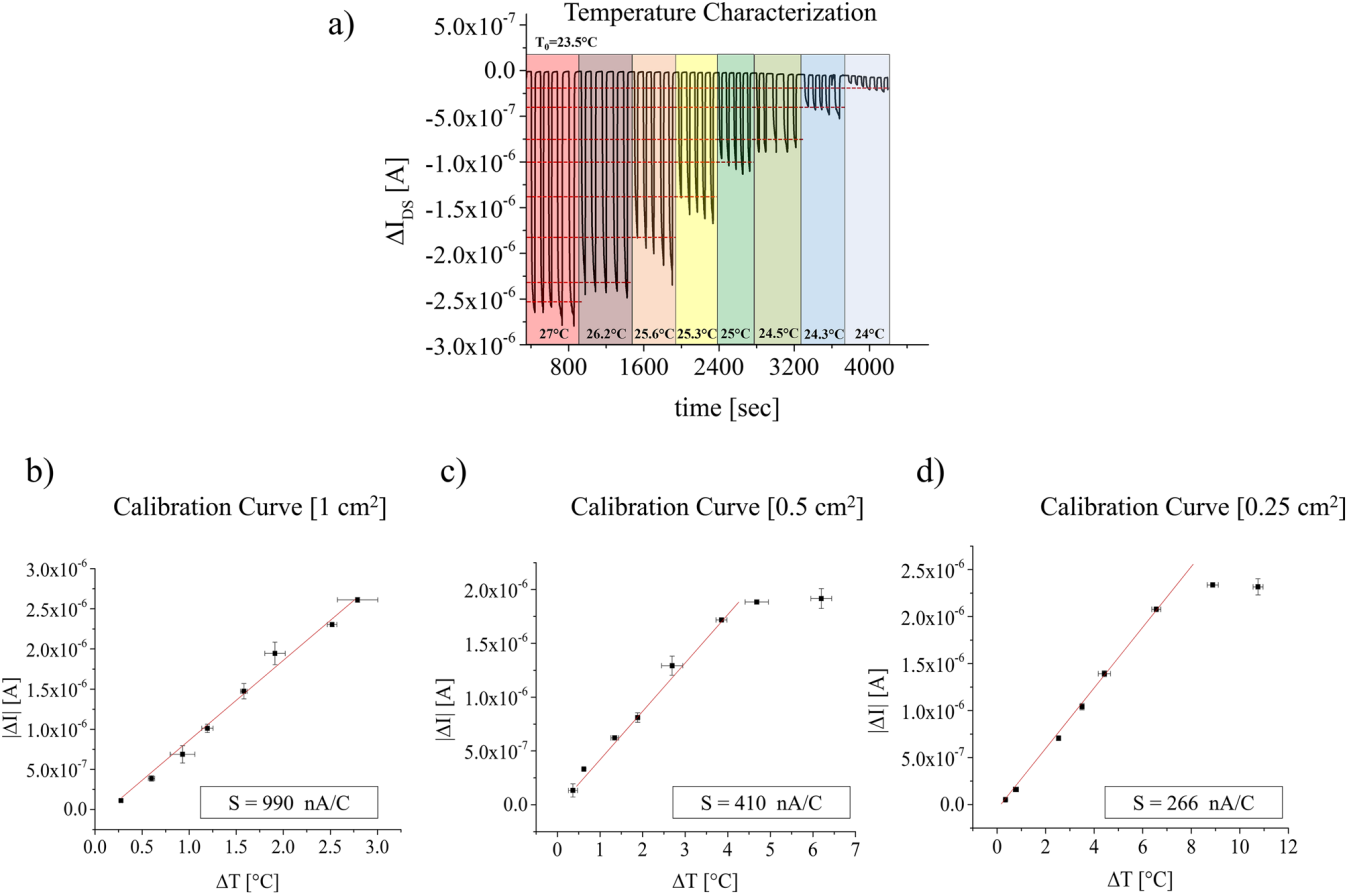
Temperature characterization for the sub-micrometer vertical channel OCMFET. (**a**) Dynamic response of the device for different temperature stimuli. In this example the sensing area, i.e. the PVDF capacitor, has an area equal to 1 cm^2^. (**b**) Calibration curve for the PVDF capacitor with an area equal to 1 cm^2^. The device shows a linear response within a 2.5 °C range and a high sensitivity about 990 nA/°C; (**c**) calibration curve for 0.5 cm^2^ and d) for 0.25 cm^2^ PVDF capacitor. As the sensing area decreases, the range of response is increased to 4–5 and 8–9 °C respectively for the curve (**c**) and (**d**). In addition, a reduction of the sensing area by a factor 2 or 4, leads also to a reduction of the sensitivity by the same factor.

Afterward, a more precise characterization of the temperature sensor has been performed (5 repetitions for each temperature value). In the first case, a 1 cm^2^ PVDF capacitor has been connected by a wire onto the floating gate. As clearly noticeable from Fig. 3a the device is capable to quickly respond to temperature variations. Moreover, as the sensitivity depends on the ratio between the sensing area (i.e. the PVDF capacitor area in this case) and the transistor area, the employment of a short channel transistor, allowed to reduce the latter thus achieving a remarkably high sensitivity around 1 μA/°C, with an average sensitivity, measured on a set of 5 different devices, of 900 ± 36 nA/°C. It is worth noting that all the devices have been fabricated in the same day, and have been characterized, intentionally, in a period of time of one month. Nevertheless, the average sensitivity did not significantly change during this period of time, as can be observed by the limited error bars reported in the plot.

Interestingly enough, we achieved a large increase in the overall sensitivity if we compare such results with what previously reported using planar OCMFETs (70 nA/°C) ^33,37^, which is also several orders of magnitude higher than what reported by Shin et al. (0.27 nA·°C^−1^)^23^ . We have performed the same analyses by connecting the same devices to different PVDF capacitors with different areas namely 0.5 cm^2^ and 0.25 cm^2^. As can be clearly observed from the plots reported in Fig. 3b–d, a reduction of the area led to an increase of the detection range eventually allowing, when using the smallest sensing area, to fit the human temperature detection range, making such devices suitable for artificial skin applications.

**Figure 4:**
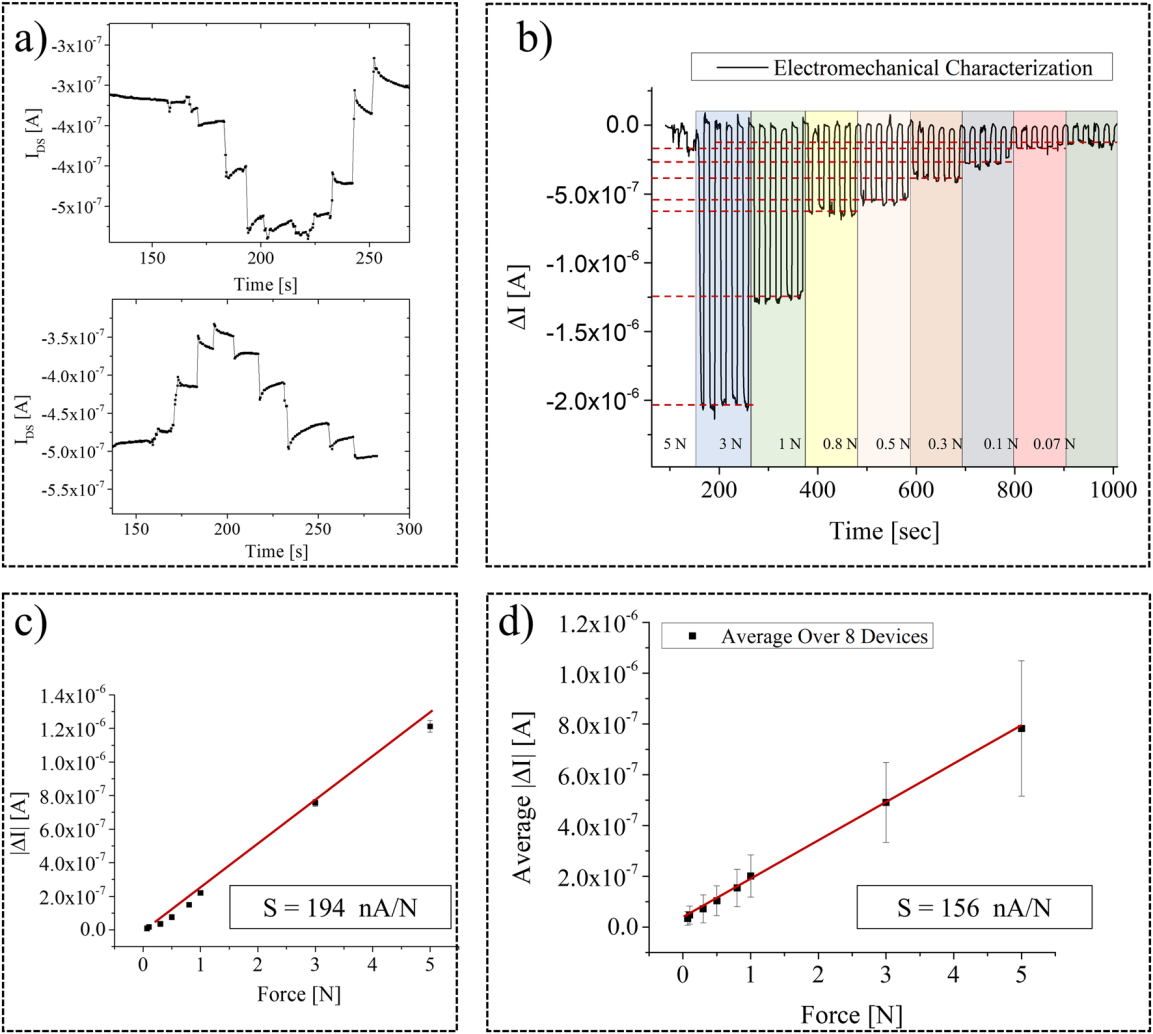
Force characterization for the proposed device. (**a**) Flipping test: connecting the top or the bottom PVDF capacitor gives rise to an opposite response. (**b**) Sensor response to different force ranges, from 5 N to 0.07 N. Each force was applied 5 times and reproducibility in sensor response is remarkable; (**c**) calibration curve of the one of the most performing devices, where the achieved sensitivity is equal to 194 nA/N; (**d**) average calibration curve, computed as the mean of the variation of the output current (mean[ΔI]), for a batch of 8 devices. The response has a linear shape in the regime 0.07–5 N and the sensitivity, i.e., the slope of the curve, is 156 nA/N.

Similarly, the same approach has been employed for the characterization of the device as force sensor. In this case, the PVDF capacitor has been placed onto a load cell and a controlled force has been exerted onto its area by means of a mechanical indenter. Also in this case, changing the connected plate of the PVDF capacitor to the floating gate, gives rise to a flipping of the sensor response, which, again, is a clear indication that the observed response is due to the piezoelectric behavior of the PVDF capacitor, as shown in Fig. 4a. In order to study the reproducibility of the fabrication process, we have set the PVDF capacitor area (1 cm^2^ for the electromechanical characterization) and we have tested a batch of 8 devices with the same geometrical parameters, i.e. the transistor channel length. Different forces, from 0.07 to 5 N five times for each value, have been applied onto the PVDF capacitor. Figure 4b, shows the force dependent current variations. Interestingly, the sensor’s response is highly reproducible, while at the same time being fully recoverable, as can be observed from the stable baseline current. Figure 4c shows the calibration curve for one of the best performing device, where the achieved sensitivity is equal to 194 nA/N, while in Fig. 4d the average calibration curve over 8 devices is plotted. the average sensitivity is 156 ± 4 nA/N, with a linear response in the range 0.07–5 N.

Also in this case, the fabricated sensors are characterized by a sensitivity which is significantly higher than what previously reported in the literature. Fastier-Wooller et al.^25^, for instance, have reported a sensitivity around 2.55% N^–1^, whereas the highest sensitivity we reported in planar sensors was around 35 nA/N^37^.

**Figure 5:**
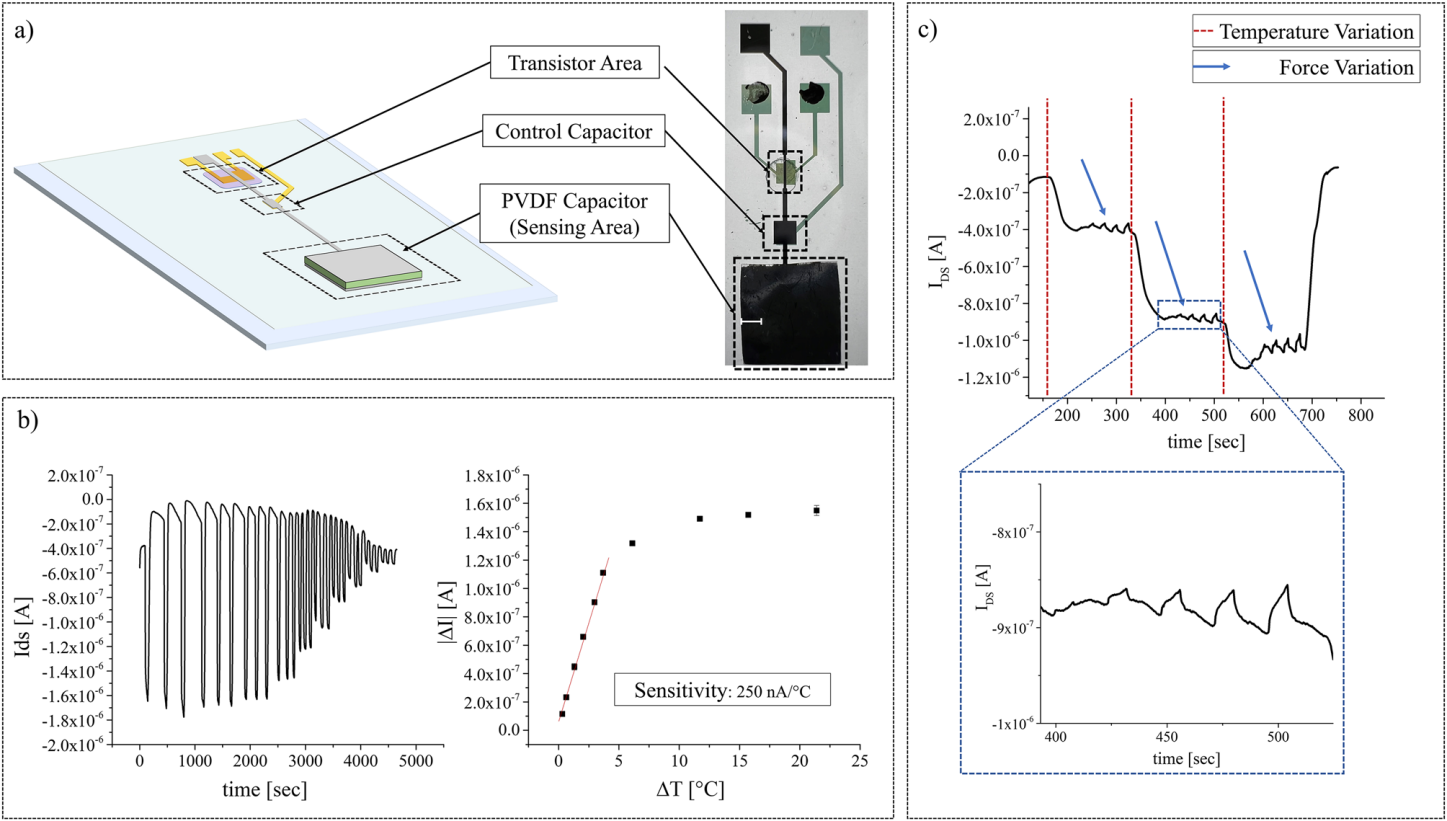
(**a**) Optical photo and sketch of the integrated vertical channel OCMFET for multimodal tactile sensing. The transistor area, the control capacitor and the PVDF capacitor glued on the floating gate are highlighted. Scale bar 1 mm. (**b**) Thermal characterization of the integrated device. The calibration curve shows a very similar results in terms of detection range and sensitivity with respect the thermal characterization reported above. (**c**) Multimodal characterization for the integrated device, where simultaneously temperature and force stimuli are exercised.

Once the devices have been independently characterized as temperature and force sensor, we have fabricated a prototype of an integrated version of the sensor, in which the PVDF capacitor, in this case with 0.25 cm^2^ area, was glued directly on the floating gate of the transistor by using a thin conductive glue layer (Fig. 5a).

At first, the device has been characterized as temperature sensor, using the same approach reported before, obtaining consistent results in terms of detection range and sensitivity (Fig. 5b).

Subsequently, the same device has been characterized as multimodal tactile sensor by inducing the simultaneous variation of temperature and force. As shown in Fig. 5c, the sensor was capable to clearly detect different applied forces for all the different temperatures. Moreover, the opposite variation of the current induced by the two stimuli allows a straightforward discrimination of the two, for example using a simple data processing algorithm. The calibration curve for the electromechanical characterization extrapolated from the dynamic characterization is reported in Figure SI 2 in the Supporting Information.

## Conclusion

In this work, we presented a highly reproducible, simple and effective strategy for the fabrication of a flexible multimodal temperature and force sensor based on an organic transistor coupled with the pyro/piezoelectric polymer poly-vinylene difluoride (PVDF). In particular, thanks to a low-resolution, and up-scalable method it was possible to obtain a sub-micrometer vertical channel OCMFET with high sensitivity and a linear response in a wide range of temperature and force variations, while reducing at the same time its overall dimensions. Moreover, we fabricated an integrated version of the device that was characterized as a multimodal sensor, showing the possibility to simultaneously transduce temperature and force stimuli. Ultimately, this innovative approach provides an interesting solution to the problem of device integration and at the same time offers a simple way to increase the sensitivity, thus opening up new possibilities for the fabrication of high spatial resolution arrays of highly sensitive organic transducers.

### Supplementary Information

Supplementary Figures.
