# Supplementary material for: Multimodal force and temperature tactile sensor based on a short-channel organic transistor with high sensitivity
Source: Sci Rep. 2023 Sep 27;13:16232. doi: 10.1038/s41598-023-43360-y (PMC10533849; doi:10.1038/s41598-023-43360-y)
Supplement: Supplementary file 1 — Supplementary Figures. [file 41598_2023_43360_MOESM1_ESM.pdf]

Multimodal force and temperature tactile sensor based on a short-channel organic transistor with high sensitivity

*Antonello Mascia, Andrea Spanu, Annalisa Bonfiglio and Piero Cosseddu**

**Corresponding authors*

A. Mascia, Prof. A. Bonfiglio, and Prof. P. Cosseddu
Department of Electrical and Electronic Engineering, University of Cagliari, via Marengo, Cagliari, Italy

A. Spanu, Prof. A. Bonfiglio
Department of Science, Technology and Society, Scuola Universitaria Superiore IUSS, Palazzo del Broletto, Piazza della Vittoria 15, 27100 Pavia, Italy

E-mail: piero.cosseddu@unica.it

Keywords: Multimodal tactile sensor, force, temperature, flexible electronics

Results

In Figure S1 the calibration curve for the electromechanical characterization of the multimodal tactile sensor is reported. The sensor was capable to clearly detect different applied forces (0.5, 1, 3, and 5 N) for all the different temperature variations when simultaneously temperature and force were excited.

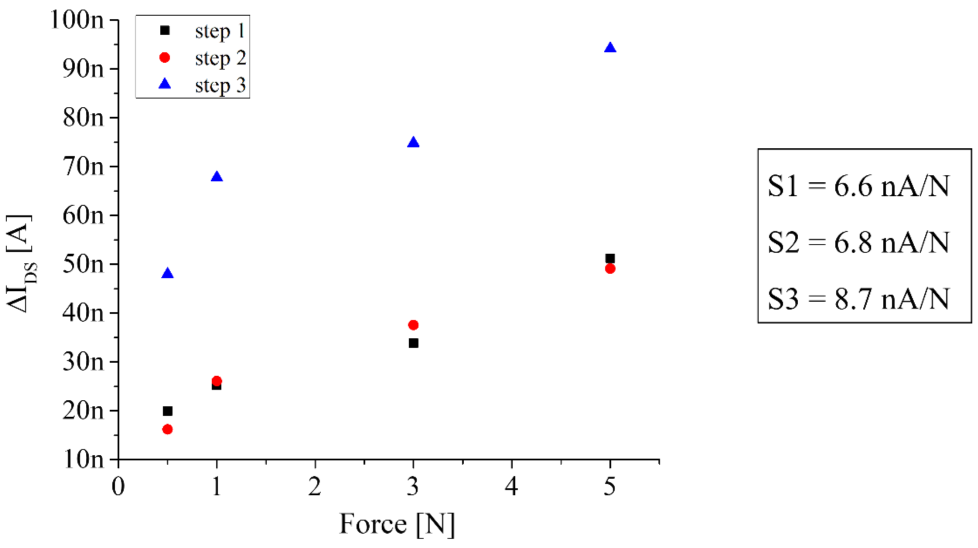

Figure S1 Calibration curve and sensitivity of the force response from the multimodal characterization.

From the transfer curves of the same device acquired at different times (the black curve refers to the measurement performed 6 months after the fabrication of the device), it is possible to notice the effect on the device structure of the time. As shown in figure S2, the device has no hysteresis and a slight reduction of the mobility and a shift of the threshold voltage, due to the ageing of the organic semiconductor, can be observed.

.

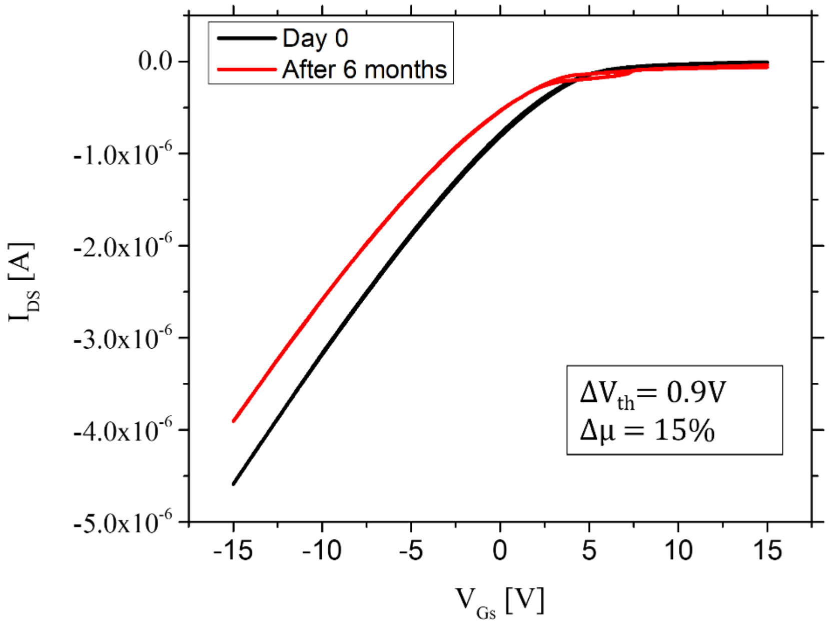

Figure S2 Transfer curves of the submicrometer vertical channel OCMFET . The black curve refers to the measurement performed on day 0, while the red curve to the measurement after 6 months from the fabrication.
